# The gut microbiome and degradation enzyme activity of wild freshwater fishes influenced by their trophic levels

**DOI:** 10.1038/srep24340

**Published:** 2016-04-13

**Authors:** Han Liu, Xianwu Guo, Ravi Gooneratne, Ruifang Lai, Cong Zeng, Fanbin Zhan, Weimin Wang

**Affiliations:** 1 College of Fisheries, Key Lab of Freshwater Animal Breeding, Ministry of Agriculture, Key Lab of Agricultural Animal Genetics, Breeding and Reproduction of Ministry of Education, Huazhong Agricultural University, Wuhan 430070, P. R. China; 2 Laboratorio de Biotecnología Genómica, Centro de Biotecnología Genómica, Instituto Politécnico Nacional, Boulevard del Maestro esquina Elías Piña, Colonia Narciso Mendoza, Ciudad Reynosa, 88710, Tamaulipas, Mexico; 3 Faculty of Agriculture and Life Sciences, Lincoln University, Lincoln 7647, New Zealand; 4 Collaborative Innovation Center for Efficient and Health Production of Fisheries in Hunan Province, Changde 41500, China

## Abstract

Vertebrate gut microbiome often underpins the metabolic capability and provides many beneficial effects on their hosts. However, little was known about how host trophic level influences fish gut microbiota and metabolic activity. In this study, more than 985,000 quality-filtered sequences from 24 16S rRNA libraries were obtained and the results revealed distinct compositions and diversities of gut microbiota in four trophic categories. PCoA test showed that gut bacterial communities of carnivorous and herbivorous fishes formed distinctly different clusters in PCoA space. Although fish in different trophic levels shared a large size of OTUs comprising a core microbiota community, at the genus level a strong distinction existed. Cellulose-degrading bacteria *Clostridium*, *Citrobacter* and *Leptotrichia* were dominant in the herbivorous, while *Cetobacterium* and protease-producing bacteria *Halomonas* were dominant in the carnivorous. PICRUSt predictions of metagenome function revealed that fishes in different trophic levels affected the metabolic capacity of their gut microbiota. Moreover, cellulase and amylase activities in herbivorous fishes were significantly higher than in the carnivorous, while trypsin activity in the carnivorous was much higher than in the herbivorous. These results indicated that host trophic level influenced the structure and composition of gut microbiota, metabolic capacity and gut content enzyme activity.

Vertebrate gut microbiome is a complex microbial ecosystem containing diverse and abundant bacteria, archaea, and fungi. These gut microbial communities reinforce the metabolic capacity and provide a series of beneficial effects on their hosts, such as nutrient digestion, immune function, and protection from invasive pathogens^1^,^2^,^3^. No endogenous genes coding cellulose-digesting enzymes were found in the genome of mammals^4^, but some putative cellulose-digesting microbes in *Clostridium* group I exist in the host fecal samples^5^. Certain gut microbiota was able to metabolize a remarkable variety of substrates, including fibrins in diets. Some typical microbial species belonging to *Aeromonas, Enterobacter, Citrobacter, Bacillus,* and *Pseudomonas* isolated from the gastrointestinal tract of herbivorous fish species were identified as the cellulolytic enzyme-producing bacterial community^6^,^7^.

It is well recognized that the structure and composition of vertebrate gut microbiota and their ecological function is strongly influenced by a range of factors that include the host genetics, living environment, diet, and phylogeny^8^,^9^,^10^,^11^. A study on the mammals indicated that host diets strongly influenced their gut bacterial diversity, which increased as the host animal diet changed from carnivorous to omnivorous, to herbivorous^9^. A short-term macronutrient change experiments in humans also showed that animal-based diet increased the abundance of bile-tolerant microorganisms and decreased the plant polysaccharides-decomposing related bacteria^3^. An early study in herbivorous surgeonfish demonstrated that the distinctive and diverse gut microbiota composition is closely associated with host trophic level^8^. Thus, diet category or host trophic level is the major factor driving the composition and metabolism of gut microbiota. In addition, environment and rearing density, temperature, and sampling time also play significant roles in establishing animal gut microbiota. In silver carp and gizzard shad, the environmental location and sampling time strongly influenced the composition of the intestinal microbiota^12^. Even in the same fish species, the gut microbiota diversity varies when reared in different environments. The most abundant gut microbiota of grass carp collected from artificial ponds near the middle reaches of Yangtze River (Wuhan, China) were dominated by *Proteobacteria*, *Firmicutes*, *Cyanobacteria*, and *Actinobacteria*, respectively, while grass carps from Dongxihu Fish Farm (Wuhan, China) were dominated by *Fusobacteria*, *Firmicutes*, *Proteobacteria*, *Bacteroidetes*^13^,^14^. This suggests that composition and diversity of animal gut microbiota is influenced by many, not independent, factors. Different niches have not the same availability of diet, which really affects the base for comparison of gut microbiota between animal species.

In the past decades, most studies investigated the gut bacterial diversity under laboratory conditions by using isolation and cultivation approaches, PCR denaturing gradient gel electrophoresis (DGGE)^15^ and terminal restriction fragment length polymorphism (T-RFLP)^16^. Next-generation sequencing of 16S rRNA gene as a culture-independent molecular techniques have greatly expanded the ability to obtain more comprehensive and complexity microbial community^17^,^18^,^19^. Further, high-throughput DNA sequencing has been used to explore the gut microbiota composition of some commercially viable fishes, including European sea bass^20^, grass carp^13^,^14^, perch^21^, channel catfish^22^ and rainbow trout^23^. However, most of them were studied in the rearing conditions and very little is known about the difference in the composition of gut microbiota between fish species with distinct trophic levels from natural environments.

The main objective of this work was to explore the diversities and complexities of gut communities in wild fish species with different trophic levels and assess the potential microbial roles in food digestion. We utilized a meta-analysis of 16S rRNA gene sequence for comparison. To minimize the influence of environmental factors, fish belonging to eight species in four trophic levels (herbivorous, carnivorous, omnivorous, filter-feeding) were collected at the same time point and in the same water area. Meanwhile, we further determined the gut content cellulase, amylase, trypsin enzyme activities of these eight species. The present study clarifies the importance of gut microbiota in digestion and provides evidence to understand how host trophic levels influence the composition and metabolic capacity of gut microbiota and gut content enzyme activities.

## Results

### Microbial complexity of fish gut flora

**Figure 1:**
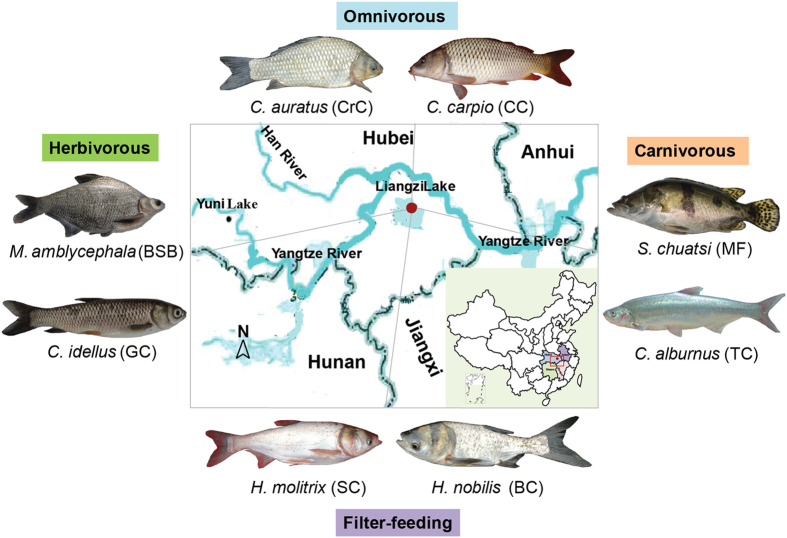
Map of the Liangzi Lake. Eight species are herbivorous *M. amblycephala* and *C. idellus*, carnivorous *S. chuatsi* and *C. alburnus*, omnivorous *C. carpio* and *C. auratus*, and filter-feeding *H. molitrix* and *H. nobilis*. The fish photos were photographed by Weimin Wang and the map was generated by QGIS 2.8 (http://www.qgis.org/en/site/), and then modified by Adobe Illustrator CS5 by Cong Zeng.

To characterize the microbial community structure of fish with different trophic levels, high-throughput sequence analysis of bacterial hyper-variable V4 region of the 16S rRNA was conducted on wild adult eight fish species captured from the Liangzi Lake, China (Fig. 1). The sampling variables including date of collection, location, water temperatures were provided in the Supplementary Table S1. A total of 985,356 quality-filtered sequences obtained from the 24 samples, ranging from 65,513 to 237,926, resulted in identification of a total of 7,349 OTUs with ≥97% sequence similarity of 53 bacterial phyla. The microbial complexity in eight fish species was estimated on the basis of alpha-diversity (the OTU number, Chao1, and Shannon index) and showed distinct differences (Table 1). BC samples had the largest alpha-diversity indices, followed by CC, and BSB samples. GC sample had the lowest alpha-diversity indices. It should be noted that alpha-diversity indices exhibit obvious difference even in the same trophic level. Based on the rarefaction curves shown in Supplementary Fig. S1, similar trend in the microbial diversity was observed in all 24 specimens, approaching the saturation plateau, except in the BSB3 and CC3 communities, which indicated incomplete sequencing efforts for the two fish samples.

### Comparison of bacterial community in fish gut

**Figure 2:**
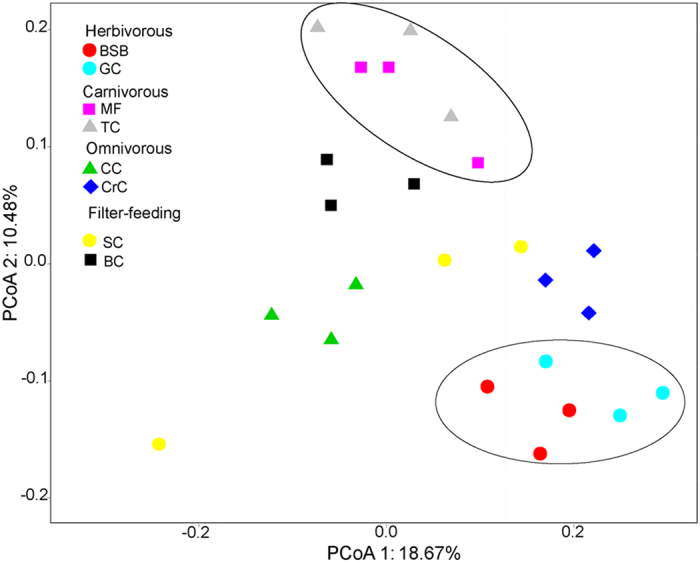
Principal coordinates analysis (PCoA) of bacterial community compositions in fish gut based on the unweighted UniFrac distance matrix. The individual samples are color-coordinated according to the fish species. BSB, red circle; GC, light blue circle; MF, purple square; TC, grey triangle; CC, green triangle; CrC, blue square; SC, yellow circle; BC, black square.

A principal component analysis (PCoA) was used to compare the similarity in the microbial community composition of 24 specimens. A scatter plot based on PCoA scores showed a clear separation of the community composition among four trophic levels fish samples (Fig. 2). The herbivorous BSB and GC samples formed a cluster and distinctly separated from the cluster of carnivorous MF and TC samples while others were located in the middle of them, indicating that this clustering pattern was influenced by their trophic levels. However, PCoA1 and PCoA2 only explained 18.67% and 10.48% of total variance respectively. It could imply the existence of other factors affecting the fish intestinal bacterial community or much finer taxonomic analysis needed.

**Figure 3:**
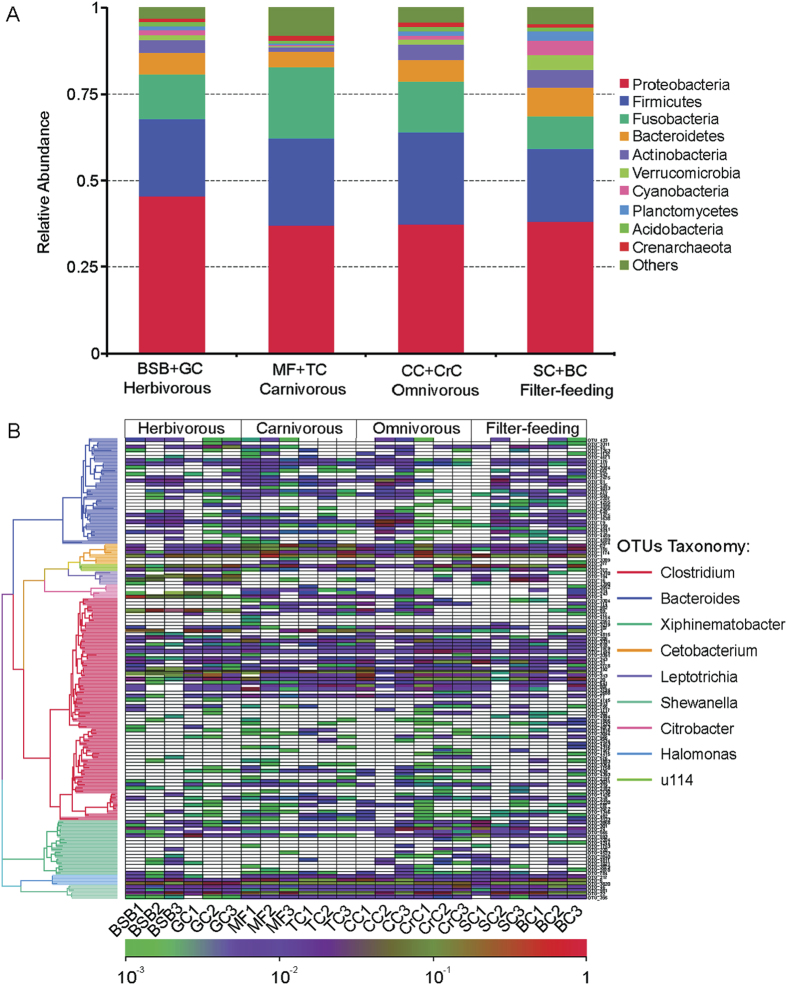
Composition of gut microbiota in fishes with four trophic levels at phylum and genus level. (**A**) Each bar represents average relative abudance of each bacterial taxon (top 10 taxa) within a group at phylum level. (**B**) Heat map shows the relative percentage of each bacterial genus and the relative values for bacterial genus are depicted by color intensity with the legend indicated at the bottom of the figure.

Approximately 99% of the total bacteria abundance was classified into 53 phyla. The most abundant taxa (top 10) of bacteria at phylum level were shown in Fig. 3A, while the rest of the less frequent taxa were categorized as ‘others’. The most abundant phylum was *Proteobacteria* in all samples, accounting for 45.52% (in herbivorous fish species), 32.82% (in carnivorous fish species), 37.32% (in omnivorous fish species), 38.13% (in filter-feeding) of the total bacterial sequences, indicating that most quantity of gut bacterial species are from this taxon. Within the *Proteobacteria*, each fish microbiota was composed of mostly *Gammaproteobacteria*, followed by *Betaproteobacteria* and *Alphaproteobacteria*. *Firmicutes* was the second most common phylum, accounting for 22.38%, 21.83%, 27.13%, 21.16% in herbivorous, carnivorous, omnivorous, and filter-feeding species, respectively (Fig. 3A). The gut microbiota of carnivorous fishes possessed a significantly greater abundance of the phylum *Fusobacteria* (21.91%) compared with filter-feeding fishes (9.41%). Filter-feeding fishes possessed a significantly greater abundance of the phylum *Acidobacteria* (5.21%) in their gut microbiota compared with carnivorous species (1.08%). Other common taxa were *Bacteroidetes, Actinobacteria, Verrucomicrobia, Cyanobacteria, Planctomycetes, Acidobacteria, Crenarchaeota* ranging between 0.89% and 8.26% in all the experimental species. *Cyanobacteria* showed highest abundance in the filter-feeding group compared with other three groups. In Supplementary Fig. S2, a detailed profile of individual fish sample is illustrated at the phylum level.

When representative bacteria were classified into genera, a strong distinction emerged between species and even within a species. For instance, the abundance with large variation was observed in the genera of *Clostridium* (0.49% to 27.34%) in the three replicates of CC and *Cetobacterium* (1.62% to 54.77%) in the three replicates of MF samples (Fig. 3B). As shown in Fig. 3B, the most abundant (top 9) gut microbiome in all fish samples at genus level were dominated by *Clostridium* (56 OTUs)*, Bacteroides* (31 OTUs)*, Xiphinematobacter* (16 OTUs)*, Cetobacterium* (6 OTUs)*, Leptotrichia* (5 OTUs)*, Shewanella* (4 OTUs)*, Citrobacter* (4 OTUs)*, Halomonas* (3 OTUs)*, and u114* (2 OTUs). Specifically, the herbivorous and omnivorous fish groups harbored a greater proportion of *Clostridium*, in comparison with carnivorous group. However, the carnivorous species were enriched with *Cetobacterium* and *Halomonas*, while the herbivorous fish were enriched with *Citrobacter* and *Leptotrichia*. Interestingly, the omnivorous and filter-feeding fishes were enriched with both cellulose-degrading bacteria *Clostridium* and protease-producing bacteria *Halomonas*, *Cetobacterium*. The abundance of *Bacteroides* and *Shewanella* were similarly distributed in all fish samples.

### Shared and unique gut microbial populations

**Figure 4:**
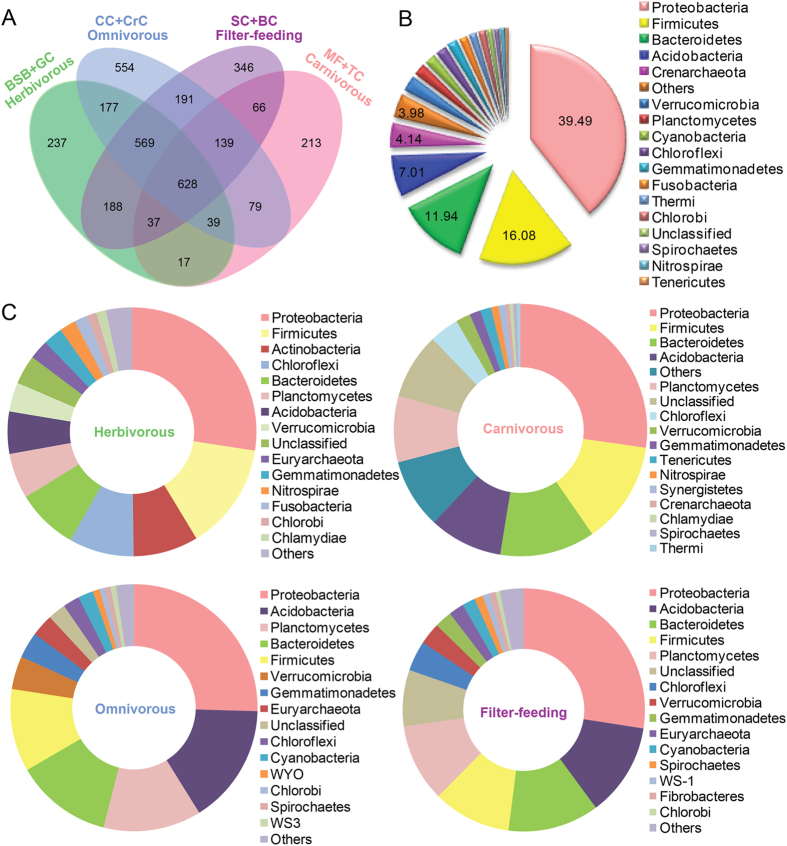
Unique and shared OTUs in fish samples with four trophic levels. (**A**) Venn diagram displays the number of shared and unique OTUs among eight fish species in different trophic levels at 30% cutoff level. (**B**) Pie chart shows the characteristic of shared OTUs with a frequency higher than 1%. (**C**) The characteristics of unique OTUs from four trophic levels with a frequency higher than 1%.

A Venn diagram was used to show the shared or unique OTUs. As shown in Fig. 4A, a total of 1,892, 1,218, 2,276, 2,164 OTUs were observed on herbivorous, carnivorous, omnivorous, and filter-feeding fish species, respectively. Interestingly, the herbivorous and carnivorous samples shared 721 OTUs, while omnivorous and filter-feeding species shared 1,527 OTUs. Carnivorous fish samples shared the least with the other feeding habit species and also exhibited the lowest number of unique OTUs, while omnivorous fish samples shared a higher number of OTUs and unique OTUs. More than 18.05% of all OTUs were shared (628 of 3480 OTUs) by four trophic levels fish species. As shown in Fig. 4B, the most abundant shared OTUs at the phylum level were *Proteobacteria* (248 OTUs, 39.49% of the total shared OTUs), *Firmicutes* (101 OTUs, 16.08%), *Bacteroidetes* (75 OTUs, 11.94%) and *Acidobacteria* (44 OTUs, 7.01%). The numbers of unique OTUs in the gut content of herbivorous, omnivorous, carnivorous and filter-feeding fish species were 237, 213, 554 and 346, respectively. These unique gut bacteria represented 12.53, 17.49, 24.34 and 15.99% of total OTUs in each of fish species. The most abundant phyla of the unique OTUs from herbivorous were Proteobacteria (27%), Firmicutes (14%), Actinobacteria (9%), Chloroflexi (8%), Bacteroidetes (8%) and others (34%), while from other three feeding habits were Proteobacteria, Firmicutes, Bacteroidetes, Acidobacteria and Planctomycetes and others with a small difference in percentage (Fig. 4C).

### Cellulose-degrading bacteria analysis

Distinct and diverse putative cellulose-degrading bacterial communities were identified in all fish samples. Interestingly, diversity of potential cellulolytic microbes was relatively high, and a number of OTUs also had a high sequence similarity to those known cellulolytic species. As shown in Supplementary Table S2, a total of 7.47% and 7.63% cellulose-degrading bacteria at the genus level were identified in herbivorous BSB and GC, respectively, while a relatively low proportion was found in carnivorous MF (2.20%) and TC (2.15%). In omnivorous and filter-feeding fish species, the cellulolytic bacterial species ranged from 4.19% to 5.27%. Among these cellulolytic bacteria, the most abundant was *Clostridium* existed in all fish species, but more frequent in the herbivorous BSB (accounting for 4.88%) and omnivorous CrC (accounting for 4.33%). In addition, *Citrobacter* and *Streptococcus* with a high proportion were also found in herbivorous BSB and GC, while *Citrobacter* was much lower and no *Streptococcus* was found in carnivorous MF and TC. *Paenibacillus*, an important cellulose-degrading bacterium, was only detected in herbivorous BSB.

**Figure 5:**
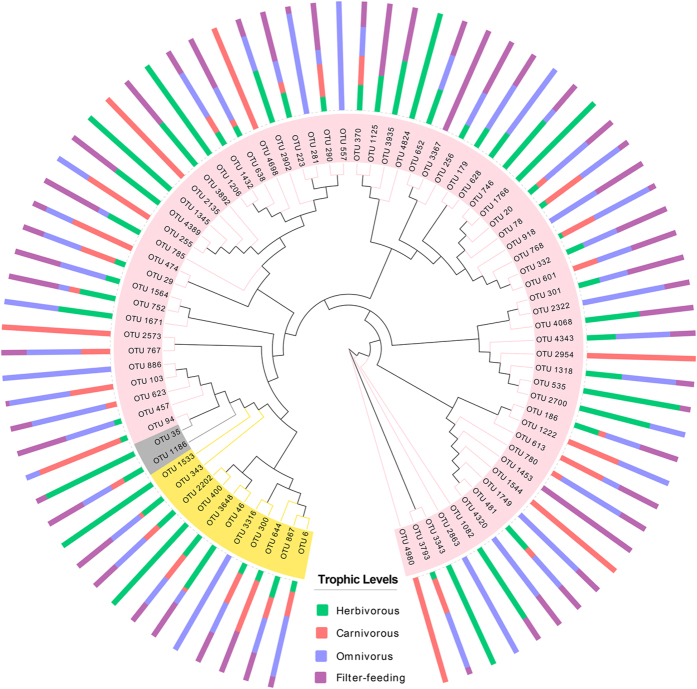
Dendrogram of cellulose-degrading represented OTUs and their host occurrence patterns. Bars show the proportion of fish samples with different trophic levels in which the given OTUs is present. Circles indicate the phylogenetic relationship of 13 kinds of cellulolytic species.

To better visualize the OTUs diversity of cellulose-degrading bacteria with a broader evolutionary context in all different feeding-habit fish species, we constructed a maximum likelihood phylogeny of all representative cellulolytic species. As shown in Fig. 5, a total of 79 OTUs were identified as 13 different cellulolytic species. Among these OTUs, 39 were classified as *Clostridium* and 19 as *Ruminococcus* which differently distributed in the four feeding-habit fish species. Most OTUs occurred in all groups with different richness. However, 5 OTUs (OTU3316, 886, 557, 918, 2863) occurred only in carnivorous species, 7 OTUs (OTU400, 1533, 1345, 1206, 4824, 1766, 3343) only in herbivorous, 6 (OTU2573, 4698, 2135, 2954, 1544, 4980) in carnivorous, and 2 (OTU3367, 1453) in filter-feeding species.

### Predicted gut microflora function using PICRUSt

**Figure 6:**
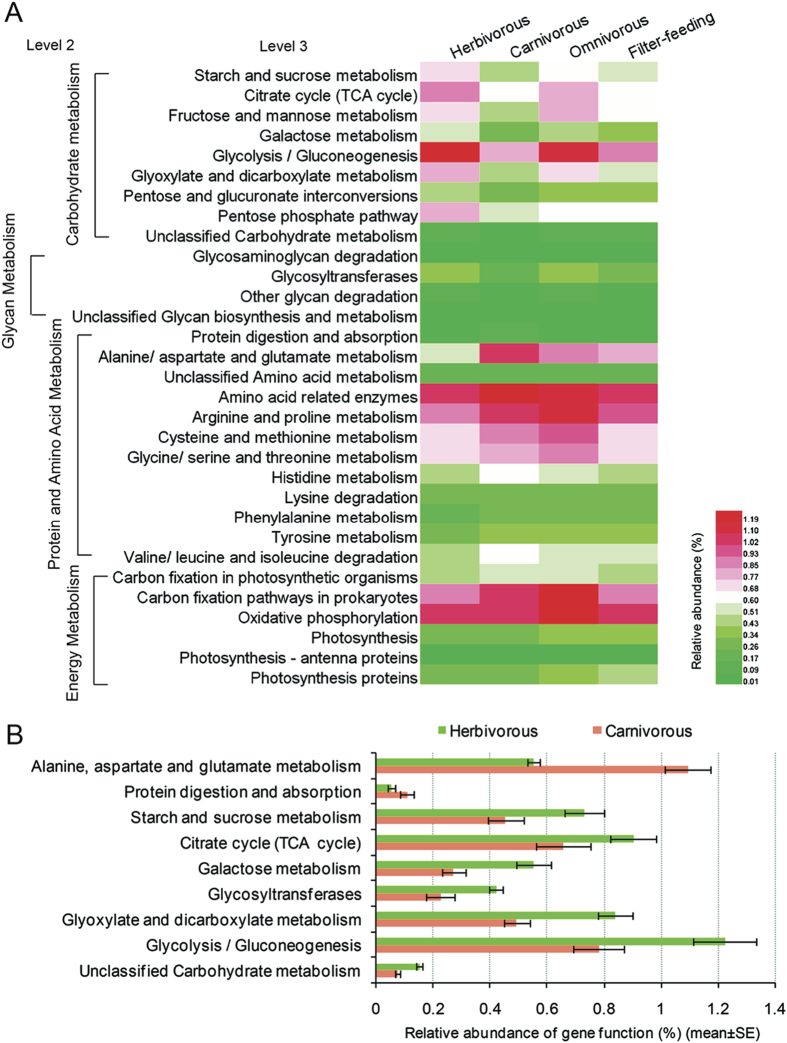
Comparison in the relative abundance of PICRUSt-generated functional profile of gut microbiota among four trophic levels. (**A**) Heat map shows the relative abundance changes in fishes with four trophic levels. (**B**) Significant differences in gene categories at level 3 (*t*-test, *P* < 0.05) between the herbivorous and the carnivorous.

PICRUSt was performed to predict the fish gut microbiome functions, which showed that four fish species in different trophic levels exhibited similar gene functions at level 2, including glycan, protein, energy and amino acid metabolism, but with some difference in abundance (Fig. 6A). The abundance of carbohydrate-related metabolism such as starch and sucrose, fructose and mannose, galactose and glycolysis/gluconeogenesis were higher in herbivorous and omnivorous fishes than in carnivorous and filter-feeding fish species. Moreover, nine gene categories showed statistically significant differences (*P* < 0.05 by *t*-test) between the herbivorous and the carnivorous as shown in Fig. 6B. This analysis allowed us to better understand the relationship between the host trophic level and metabolic capacity.

### Relationship between gastrointestinal microbiota and gut content enzyme activity

**Figure 7:**
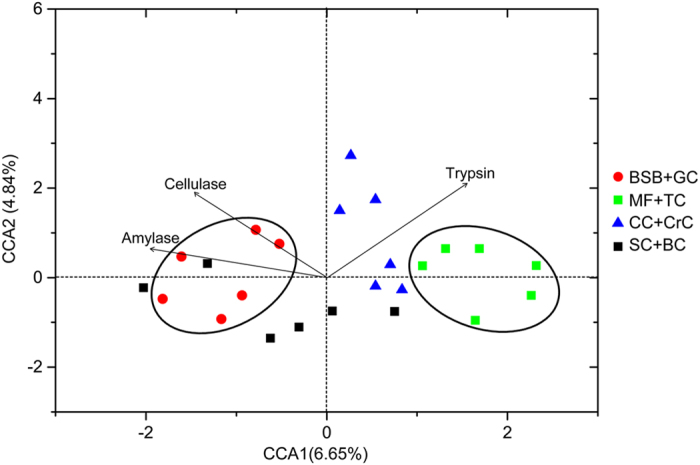
Canonical correspondence analysis (CCA) showing the correlation between the gut microbial compositions of eight fish species and their enzyme activities.

As shown in Table 2, the gut content digestive enzyme activity differed markedly in the eight fish species. Cellulase activity was significantly higher (*P* < 0.05) in herbivorous species than in carnivorous, but no obvious difference (*P* > 0.05) with omnivorous and filter-feeding fish species was detected. The amylase activity in herbivorous fish was not significantly different to the omnivorous but was 12-fold higher than in carnivorous fish. On the contrary, the trypsin activity in carnivorous was higher than in herbivorous and filter-feeding fish species (*P* < 0.05). As shown in Fig. 7, the gut microbial composition of each fish species was closely related to their metabolic enzymes. The gut microbiota composition of carnivorous species (MF and TC) were more related to trypsin activity, and estranged from cellulase and amylase activities. In contrast, the gut microbial compositions of herbivorous fish (BSB and GC) were correlated with cellulase and amylase activities.

## Discussion

It is widely acknowledged that the vertebrate gut microbial communities play critical roles in host immune system and digestive system^1^,^2^, which is now attracting increasing attention in the fish research. Previous studies have demonstrated that the variations in fish gut microbiota diversity and structure is mostly due to the dietary input^8^,^21^,^24^,^25^ and environmental locations^26^,^27^. However, most of these studies are often to characterize the gut microbiota associated with fish and limited to analysis of one or two fish species with their trophic levels. It is still little known about the information on the comparison of fish gut microbiota with several trophic levels in several fish species collected from the same water area.

Herbivorous *M. amblycephala* and *C. idellus*, carnivorous *S. chuatsi* and *C. alburnus*, omnivorous *C. carpio*, and *C. auratus*, and filter-feeding *H. molitrix* and *H. nobilis* are the most important freshwater fish species for aquaculture in China and accounted for the majority of aquatic products. To address the shortcomings of previous studies, we analyzed and compared the gut microbiota structures and gut content enzymes activities of above-mentioned wild fish species to explore the potential relationship between host trophic level and gut microbiota composition. Our results clearly indicated that trophic level dramatically affected the fish gut microbiota diversity and composition, particularly in the herbivorous and carnivorous fishes, even though they were sampled from the same environment and on the same date. Moreover, the enzyme activities including amylase, cellulose, and trypsin in gut content were also significantly different in each trophic group. Our results provide an understanding how fish trophic levels influence the gut microbiota diversity and how the gut microbiota contributes to host’s fitness and food digestion.

Principal coordinate analysis (PCoA) revealed that gut bacterial communities from the fish in different trophic levels formed different clusters. Carnivores (MF and TC), and herbivores (BSB and GC) formed distinctly clusters in PCoA space (Fig. 2), suggesting that the enrichments and diversity of gut microbiota are affected by the trophic level. This result is similar to the research of Sullam *et al.*^27^ that trophic level and host phylogeny are related to the composition of fish gut bacteria. At the phylum level, around 99% of the total bacterial abundance was classified into total 53 phyla. Among these phyla, *Proteobacteria, Firmicutes, Fusobacteria, Acidobacteria* were dominant in the eight fish species samples (Fig. 3A). This is agreement with previous studies^14^,^27^,^28^. They demonstrated that *Proteobacteria* was the most abundant phylum in many marine or freshwater fishes, and many different fish species harbored similar gut bacteria, suggesting that core gut microbiota communities might be common across a broader range of fish species. However, when representative bacteria were classified into genera, a strong distinction emerged between species and within each trophic level. In our study, *Clostridium, Citrobacter* and *Leptotrichia* were the most abundant bacteria in the herbivorous fish, while the carnivorous fish species were enriched with *Cetobacterium* and *Halomonas* (Fig. 3B). The omnivorous and filter-feeding fishes were enriched with *Clostridium*, *Cetobacterium* and *Halomonas*. Similarly, Wu *et al.*^13^ found that *Anoxybacillus*, *Leuconostoc*, *Clostridium*, *Actinomyces*, and *Citrobacter* were most abundant in grass carp samples. Previous studies have reported that *Clostridium, Citrobacter* and *Leptotrichia, Bacillus, Enterobacter* were the important cellulose-degrading bacteria^12^,^29^,^30^. Interestingly, the *Cetobacterium* was the most abundant species in carnivorous channel catfish and largemouth bass^22^. *Halomonas* was identified as one of the most predominant cultivated protease-producing bacteria because of its chemoorganotrophic nature^31^,^32^. It suggested that above-mentioned bacterial species might play significant roles in their host’s digestion system.

Discovering the core gut microbiome is crucial for understanding the ecology of microbial consortia and it is the first step to define a stable and healthy bacterial community in animal intestines^33^. In the present study, we found that all eight fish species in distinct trophic levels shared a large core microbiota (Fig. 4A), comprising a relative large number of OTUs (628), which was dominated by *Proteobacteria* (248 OTUs), *Firmicutes* (101 OTUs), *Bacteroidetes* (75 OTUs), and *Acidobacteria* (44) (Fig. 4B). This core microbiota might be an important gut microbiota composition. Wong *et al.*^34^ found that the intestinal microbiota of rainbow trout on different diets categories were shared by large core bacterial lineages. The factors underlying the large size of shared gut microbiota remain unknown. The eight wild fish species studied here were collected from the same Lake at same time-point. It is thus likely that large shared OTUs might be due to the shared similar environment. Previous studies in zebrafish and mice found that core microbiota in domesticated and wide animals displayed salient differences owing to their life histories and local environments^28^,^35^. In spite of this, different trophic level fish species in our study still displayed obvious differences in the proportion of these dominant microbial communities (Fig. 3), especially with strikingly different dominant bacteria at the genus level, which indicated that the fish specific endogenous factors including their trophic levels far outweighed the environmental factors to shape fish gut mibirobiota.

It has been reported that food digestion depends on the aid of gut symbiotic microorganisms to digest food and supply the energy to the host^6^. Our study highlighted the three main enzyme activities and possible contribution of gut microbiota in food digestion. Cellulase and amylase activity in the gut content of herbivorous fish species was significantly higher (*P* < 0.05) than in carnivorous fish, while trypsin activity in carnivorous fish was much higher than in herbivorous and filter-feeding fishes (Table 2). This result is consistently related to the composition of gut microbiota, clearly demonstrated by Canonical correspondence analysis (CCA) (Fig. 7). Although whether the gut content enzymes are produced by the host or by the gut microbiota is not exactly known, our study clearly indicated that host trophic levels overtly influenced their gut microbiota composition and enzyme activities. Yokoe and Yasumasu^36^ mentioned that fish does not posses endogenous cellulose and therefore cellulose digestion depends on the exogenous cellulose. Interestingly, a previous study found a diet-dependent cellulase activity both in the intestine and hepatopancreas of rohu (*Labeo rohita*) fingerlings^37^. The cellulase activity sharply decreased when the fish were fed diets containing antibiotic tetracycline suggesting that the gut cellulase activity is largely contributed by the gut microbiota. These researches indicated that some certain gut microbiota help fish to digest food.

Gut microbiota could be affected by multiple factors. Host genetic background determines the host trophic level and influences the individual gut microbiome diversity^9^,^38^. In addition, environmental locations or fish ages have effects on the microbial diversity in different feeding habit fishes. It would be particularly interesting to determine whether same wild fish species in other water bodies with similar size have different gut microbial composition. Further, how the bacteria coordinate in the gut microbiota and how these bacteria interact with their hosts need to be clarified. Thus, more topics on ecology and physiology of gut microbiota in fish are very attractive fields for study.

In conclusion, this study is the first comprehensive, high-throughput analyses of the gut microbiota diversity in up to eight wild fish species with multiple trophic levels under the same environmental conditions. Carnivorous and herbivorous fish species formed significant clusters in PCoA space suggesting that the enrichments and diversity of gut microbiota were affected by the trophic levels. Although eight fish species shared a large size of OTUs comprising a core microbiota community dominated by *Proteobacteria, Firmicutes, Bacteroidetes, Acidobacteria,* at the genus level, a strong distinction in composition of gut microbiota occurred between fish species in different trophic levels or even within a species. Herbivorous fish harbored abundant of cellulose-degrading bacteria including *Clostridium, Citrobacter* and *Leptotrichia,* while carnivorous fish species were enriched with *Cetobacterium* and protease-producing bacteria *Halomonas*. The omnivorous and filter-feeding fish were enriched with both cellulose-degrading bacteria *Clostridium*, *Leptotrichia* and protease-producing bacteria *Halomonas*, *Cetobacterium.* Moreover, cellulase and amylase activity in the gut content of herbivorous fish species were significantly higher than in the carnivorous fish. In contrast, the trypsin activity in carnivorous was much higher than in herbivorous and filter-feeding fish species. These results indicate a strong influence of the host trophic levels and phylogeny on the structure and diversity of gut microbiota.

## Methods

### Sample collection

Wild adult eight fish species were captured from the center of freshwater ecosystem of Liangzi Lake (30°50′–30°180′N, 114°210′–114°390′E), HuBei Province, China on October 10, 2013 (Fig. 1). Liangzi Lake is a mesotrophic shallow lake located on the middle reaches of the Yangtze River Basin, with typical levels of dietary and genetic diversity in the region. We captured more than 24 fishes from eight fish species classified into four trophic levels including herbivorous *Megalobrama amblycephala* (blunt snout bream, BSB) and *Ctenopharyngodon idellus* (grass carp, GC), carnivorous *Siniperca chuatsi* (mandarin fish, MF) and *Culter alburnus* (topmouth culter, TC), omnivorous *Cyprinus carpio* (common carp, CC) and *Carassius auratus* (crucian carp, CrC), and filter-feeding *Hypophthalmichthys molitrix* (silver carp, SC) and *Hypophthalmichthys nobilis* (bighead carp, BC) using a fish trap set along 500 m, approximate 3 m deep.

All the experimental procedures involving fish were performed in accordance with the guidelines and regulations of National Institute of Health Guide for the Care and Use of Laboratory Animals. The experiments were also approved by the Animal Care and Use Committee of Huazhong Agricultural University. Prior to dissection, fishes were euthanized with an overdose of tricaine methanesulfonate (dissolved in water). All procedures for handling and euthanasia of wild freshwater fish species were approved by institution animal care. To help eliminate transient bacteria, the whole intestinal tract of individual fish was dissected with sterile instruments and washed in 70% ethanol and sterile water. Then the gut content from the midgut region to the hindgut region were squeezed out and mixed thoroughly, and then collected into sterile tubes and immediately stored at liquid nitrogen.

### DNA extraction, amplification and sequencing

A 200 mg sample was taken from each tube of the fish gut content and homogenized using a three-minute bead beating procedure at 30 Hz. Bacterial genomic DNA was extracted using a QIAamp DNA Stool Mini Kit (Qiagen, Valencia, USA) following the manufacturer’s recommendations. DNA from these fish samples were extracted in a similar manner. The quality and integrity of each DNA sample was determined by electrophoresis in 1% agarose gel with Tris-acetate-EDTA (TAE) buffer. DNA concentration was quantified using NanoDrop ND-2000 spectrophotometer (Thermo Scientific). 24 libraries were constructed and sequenced using the Illumina MiSeq sequencing platform. PCR amplifications were conducted from each sample to produce the V4 hypervariable region (515F and 806 R) of the 16S rRNA gene according to the previously described methods^39^,^40^. The reverse primer contained a 6-bp error-correcting barcode unique to each sample. The sequencing was performed at Novogene Bioinformatics Technology Co., Ltd, Beijing, China.

### Taxonomic analyses of sequenced reads

All sequences have been deposited in the NCBI’s Sequence Read Archive (SRA accession number to be provided upon acceptance). FLASH software was used to merge Pairs of reads from the original DNA fragments when the original DNA fragments are shorter than twice the length of reads^41^. Raw data were filtered using the open-source software system Quantitative Insights into Microbial Ecology (QIIME) quality filters^42^,^43^. Then we used UPARSE pipeline to pick operational taxonomic units (OTUs) at an identity threshold of 97%. We picked a representative sequences for each OTU and used the RDP classifier tool^39^ to assign taxonomic data to each representative sequence.

In order to estimate individual hosts’ microbial alpha diversity, the rarefaction curves were generated based on metrics and the number of operational taxonomical units (OTUs) present in the samples was determined (Chao1 metric and shannon index). To minimize the biases caused by sequencing effort differences, equal numbers of sequences were used. The values were summarized per species of fish as the means and standard deviations (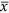 ± σ_*x*_). A 97% sequence identity of the 16S rRNA gene was used to determine OTUs and calculated the richness of Chao1 and indexes of Shannon diversity in species-level.

Core gut microbiota for each fish species was defined and analyzed by indentified the shared OTU among three replicates^33^. The shared and unique OTUs among the four trophic levels were also represented by a Scale-Venn diagram using eulerAPE (http://www.eulerdiagrams.org/eulerAPE/). The gut microbiota, beta diversity and taxon composition were analyzed by QIIME for calculating both weighted and unweighted UniFrac.

To better understand the forces that shape the fish gut composition, the eight fish species were sampled from the same area and assigned to four different diet categories (herbivorous, carnivorous, omnivorous or filter-feeding). The unweighted UniFrac phylogenetic distance metric was analyzed by using a Principal Coordinate Analysis (PCoA) and Unweighted Pair Group Method with Arithmetic mean (UPGMA) Clustering. To explore the metabolic activity of the bacterial communities found on the gut contents of different trophic level fish species, a bioinformatics tool PICRUSt (Phylogenetic Investigation of Communities by Reconstruction of Unobserved States)^44^ was used to generate the KEGG (Kyoto encyclopedia of genes and genomes) pathway. The functions were categorized at levels 2 and 3.

### Analysis of Enzyme activities

For enzymatic analysis, 200 mg sample of fish gut content was homogenized in 2 mL using a hand-held glass homogenizer and chilled in 0.1 M phosphate buffer on ice (PBS, pH 6.8, 1:20 w/v). The homogenate was centrifuged at 12,000 × *g* for 20 min at 4 °C. After centrifugation, the supernatant was divided into four Eppendorf tubes and then stored at −40 °C until analysis for use. All enzymatic assays were conducted within 3 days after extraction.

Cellulase activity was evaluated according to the method of Miller^45^ as described by Anand *et al.*^46^. Briefly, 500 μL 1% carboxymethyl cellulose (CMC) as substrate was added in 1 mL PBS (0.1 M, pH 6.8) with 500 μL sample and incubated at 37 °C for 1 h. Reaction was stopped by adding 3 mL of DNS reagent and then kept in boiling water bath for 10 min. The absorbance of reaction mixture was recorded at 574 nm. One cellulase activity unit was defined as number of molecules of glucose released from cellulose mg^−1^ protein min^−1^ at 37 °C. Amylase activity was measured by the 3,5-dinitrosalicylic acid (DNS) method modified by Rick and Stegbauer^47^ with one amylase activity unit that was defined as the number of molecules of maltose released from starch mg^−1^ protein min^−1^ at 37 °C. The reaction mixture recorded the absorbance at 540 nm. When amylase activity of the sample was too high, PBS was added to dilute the sample. Trypsin activity was determined by a modified method as described by German and Bittong^48^ and Erlanger *et al.*^49^, using Nα-Benzoyl-DL-arginine *p*-nitroanilide hydrochloride (BAPNA) (Sigma B4875) as the specific substrate. Readings were taken at 410 nm about every 15 sec for 5 min. The trypsin activity was determined from a *p*-nitroaniline standard curve and expressed as micromole of *p*-nitroaniline released mg^−1^ protein min^−1^ at 37 °C. Protein content of the supernatant was measured according to the Bradford^50^ method using the bovine serum albumin as the standard. To better understand the relationship between gut microbial diversity and its enzymes activity in fishes with different trophic levels, the canonical correspondence analysis (CCA) was conducted.

## Additional Information

**How to cite this article**: Liu, H. *et al.* The gut microbiome and degradation enzyme activity of wild freshwater fishes influenced by their trophic levels. *Sci. Rep.* **6**, 24340; doi: 10.1038/srep24340 (2016).

## Supplementary Material

Supplementary Information

## Figures and Tables

**Table 1:** Overview of fish samples including quality sequences and index of α diversity.

Fish Species	Trophic levels	Sequenced library No.	Total filtered quality sequences	Richness estimates		Diversity estimates
				OTUs	Chao1	Shannon
BSB	Herbivorous	3	140046	1135 ± 308	1813 ± 372	6.74 ± 1.07
GC	Herbivorous	3	113291	560 ± 130	932 ± 179	5.12 ± 0.51
MF	Carnivorous	3	158623	811 ± 157	1543 ± 268	4.23 ± 0.58
TC	Carnivorous	3	92677	937 ± 203	1350 ± 146	5.40 ± 0.91
CC	Omnivorous	3	65513	1202 ± 367	1526 ± 497	7.45 ± 0.96
CrC	Omnivorous	3	237926	562 ± 87	1024 ± 141	4.74 ± 0.46
SC	Filter-feeding	3	94761	927 ± 216	1379 ± 323	6.46 ± 0.75
BC	Filter-feeding	3	82519	1215 ± 151	1817 ± 241	6.91 ± 0.54

Operational taxonomic units (OTUs) are defined at 97% sequence similarity.

**Table 2:** Fish gut content enzymes activities (U/mg protein).

Trophic levels	Species	Gut content enzymes activities		
		Cellulase	Amylase	Trypsin
Herbivorous	BSB	16.55 ± 4.55^abc^	263.55 ± 52.17^a^	9.25 ± 1.69^cd^
	GC	20.08 ± 6.45^a^	252.62 ± 51.59^a^	6.83 ± 1.35^d^
Carnivorous	MF	8.69 ± 1.61^bc^	12.93 ± 2.87^c^	13.13 ± 1.17^b^
	TC	6.78 ± 0.81^c^	27.75 ± 7.69^c^	19.63 ± 2.12^a^
Omnivorous	CC	8.90 ± 1.82^bc^	222.01 ± 71.83^ab^	12.49 ± 1.98^bc^
	CrC	16.70 ± 5.16^ab^	237.67 ± 42.07^a^	15.27 ± 2.91^b^
Filter-feeding	SC	11.56 ± 1.68^abc^	354.17 ± 68.91^a^	9.56 ± 0.84^cd^
	BC	8.61 ± 1.48^bc^	93.69 ± 19.83^bc^	6.85 ± 1.12^d^

The means (mean ± SE) with different letters in each enzyme indicate significant differences. ANOVA was followed by Tukey’s test, *P *< 0.05.
