# Supplementary material for: The gut microbiome and degradation enzyme activity of wild freshwater fishes influenced by their trophic levels
Source: Sci Rep. 2016 Apr 13;6:24340. doi: 10.1038/srep24340 (PMC4829839; doi:10.1038/srep24340)
Supplement: Supplementary Information [file srep24340-s1.pdf]

The gut microbiome and degradation enzyme activity of wild freshwater fishes influenced by their trophic levels

Han Liu[1], Xianwu Guo[3], Ravi Gooneratne[4], Ruifang Lai[1], Cong Zeng[1], Fanbin Zhan[1], Weimin Wang[1,2 *]

[1]College of Fisheries, Key Lab of Freshwater Animal Breeding, Ministry of Agriculture, Key Lab of Agricultural Animal Genetics, Breeding and Reproduction of Ministry of Education, Huazhong Agricultural University, Wuhan 430070, P. R. China

[2]Collaborative Innovation Center for Efficient and Health Production of Fisheries in Hunan Province, Changde 41500, China

[3]Laboratorio de Biotecnología Genómica, Centro de Biotecnología Genómica, Instituto Politécnico Nacional, Boulevard del Maestro esquina Elías Piña, Colonia Narciso Mendoza, Ciudad Reynosa, 88710, Tamaulipas, Mexico

[4]Faculty of Agriculture and Life Sciences, Lincoln University, Lincoln 7647, New Zealand

[*]Correspondence and requests for materials should be addressed to W.M.W. (wangwm@mail.hzau.edu.cn)

Supplementary Information

Supplementary information contains two Figures and two Tables.

Figure S1

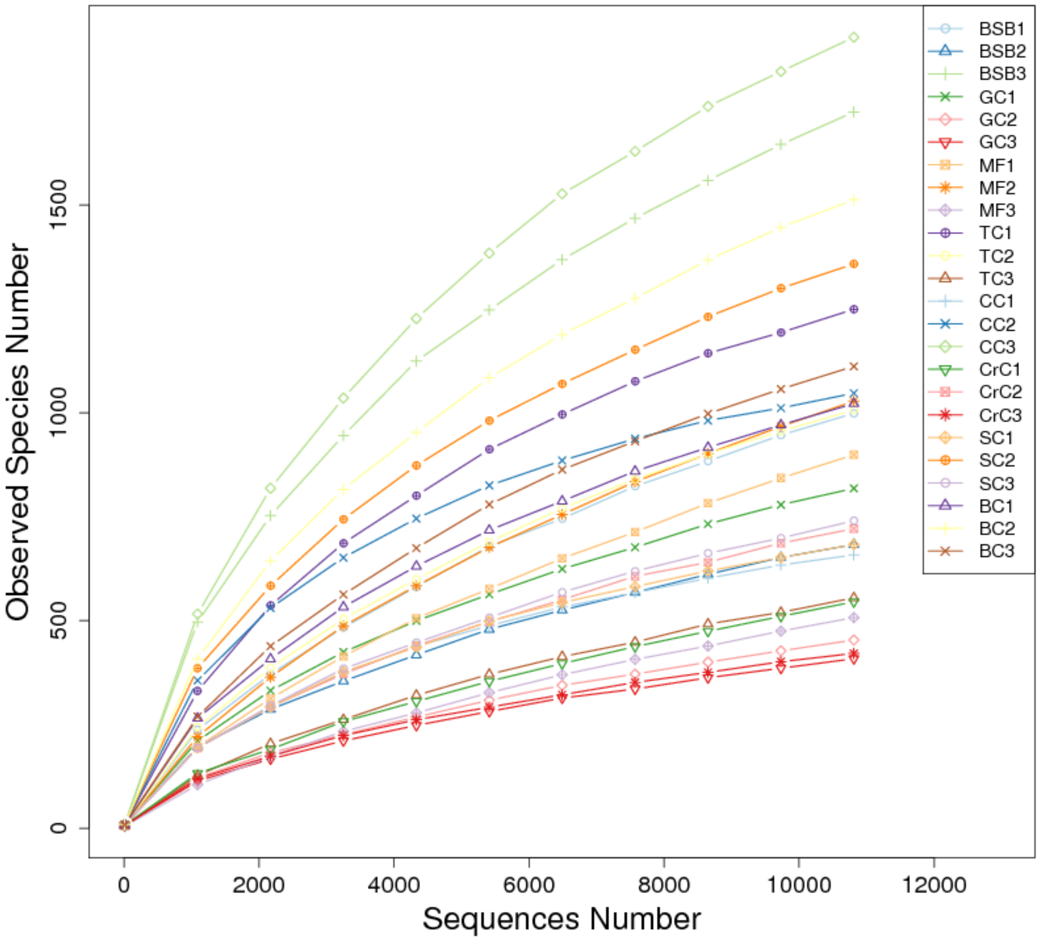

Figure S1 Rarefaction analysis of different fish gut samples. Rarefaction curves analysis showing the number of detected OTUs per sample based on sequence number for 24 specimens. Sequences are clustered with similarity cut-off of 97%.

Figure S2:

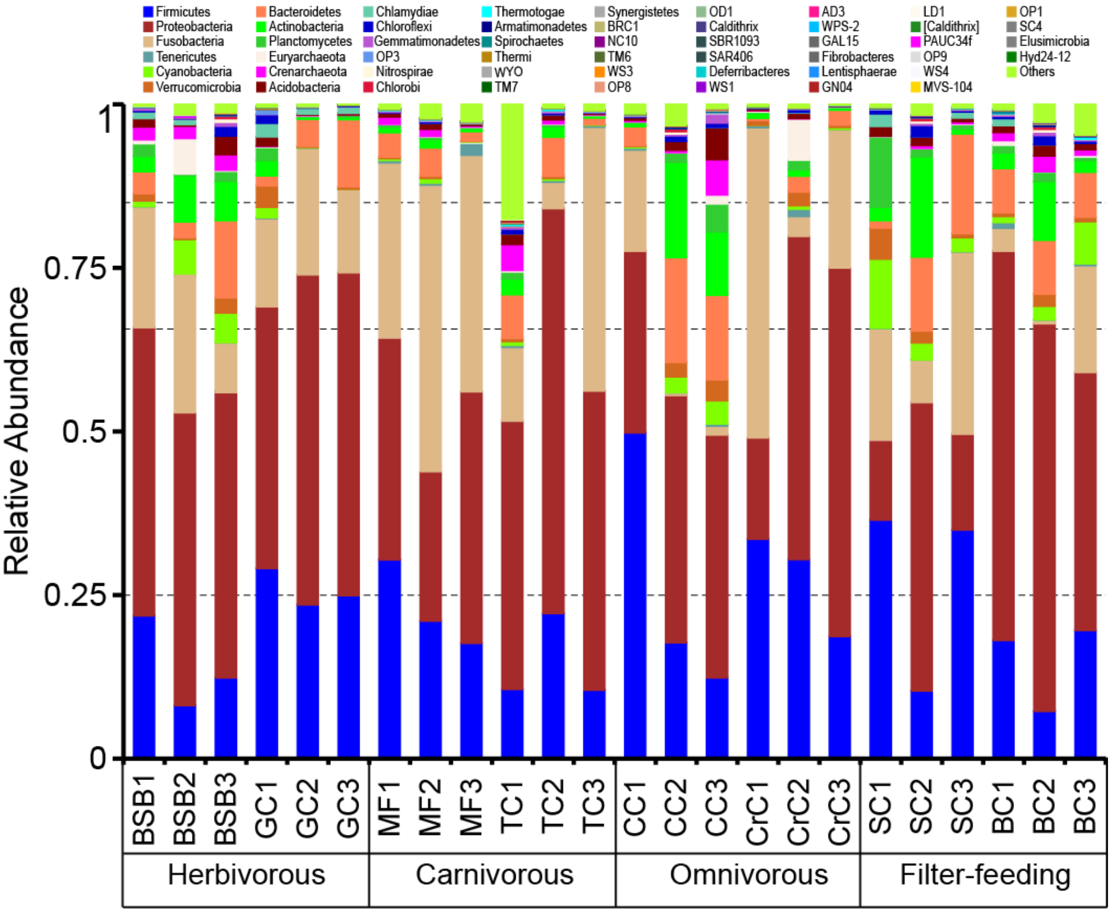

Figure S2 Individual relative abundance of OTUs for all taxa of gut microbiota in fishes with four trophic levels at phylum level.

Table S1:

Table S1 Sampling variables and statistics of sequenced raw data.

Library ID	Sample Name	Trophic levels	Rearing	Sampling Time	Temperature (℃)	Raw Data	Average Lenth (bp)	Q30	GC%
1	BSB1	Herbivorous	Wild	2013.11.6	16±0.8	34,289	254	94.56	53.21
2	BSB2	Herbivorous	Wild	2013.11.6	16±0.8	90,299	256	94.31	54.82
3	BSB3	Herbivorous	Wild	2013.11.6	16±0.8	36,467	259	94.86	54.74
4	GC1	Herbivorous	Wild	2013.11.6	16±0.8	29,834	253	94.39	53.07
5	GC2	Herbivorous	Wild	2013.11.6	16±0.8	61,080	254	94.59	52.48
6	GC3	Herbivorous	Wild	2013.11.6	16±0.8	37,230	253	94.5	51.57
7	MF1	Carnivorous	Wild	2013.11.6	16±0.8	68,241	254	94.74	53.13
8	MF2	Carnivorous	Wild	2013.11.6	16±0.8	45,989	253	94.6	50.92
9	MF3	Carnivorous	Wild	2013.11.6	16±0.8	52,235	253	94.72	49.32
10	TC1	Carnivorous	Wild	2013.11.6	16±0.8	19,060	253	93.82	54.48
11	TC2	Carnivorous	Wild	2013.11.6	16±0.8	32,373	254	94.53	54.27
12	TC3	Carnivorous	Wild	2013.11.6	16±0.8	47,665	254	94.97	51.69
13	CC1	Omnivorus	Wild	2013.11.6	16±0.8	14,287	254	94.79	52.12
14	CC2	Omnivorus	Wild	2013.11.6	16±0.8	18,958	260	94.46	54.24
15	CC3	Omnivorus	Wild	2013.11.6	16±0.8	39,124	254	94.46	54.85
16	CrC1	Omnivorus	Wild	2013.11.6	16±0.8	123,644	254	94.6	50.7
17	CrC2	Omnivorus	Wild	2013.11.6	16±0.8	64,240	254	95.55	52.67
18	CrC3	Omnivorus	Wild	2013.11.6	16±0.8	86,152	253	94.72	52.47
19	SC1	Filter-feeding	Wild	2013.11.6	16±0.8	31,144	254	93.9	53.27
20	SC2	Filter-feeding	Wild	2013.11.6	16±0.8	33,656	259	94.85	54.14
21	SC3	Filter-feeding	Wild	2013.11.6	16±0.8	43,255	253	94.66	51.62
22	BC1	Filter-feeding	Wild	2013.11.6	16±0.8	24,553	254	94.15	53.92
23	BC2	Filter-feeding	Wild	2013.11.6	16±0.8	36,404	259	95.13	54.61
24	BC3	Filter-feeding	Wild	2013.11.6	16±0.8	32,256	254	94.64	53.08

Table S2

Table S2 Relative abundance (%) of potential cellulose-degrading bacteria in different trophic levels freshwater fish gut samples.

**Bacteria**	**Herbivorous**		**Carnivorous**		**Omnivorus**		**Filter-feeding**	
	**BSB**	**GC**	**MF**	**TC**	**CC**	**CrC**	**SC**	**BC**
Vibrio	0.04	0.04	0.03	0.06	0.13	0.02	0.04	0.08
Bacillus	0.04	0.02	0.03	0.03	0.07	0.02	0.03	0.03
Actinomyces	0.02	0.01	0.00	0.01	0.01	0.00	0.01	0.02
Clostridium	4.88	3.61	1.94	1.66	2.78	4.33	3.86	2.68
Citrobacter	1.42	1.05	0.03	0.07	0.31	0.09	0.52	0.74
Erwinia	0.08	0.05	0.05	0.15	0.13	0.16	0.06	0.07
Pseudomonas	0.17	0.02	0.05	0.07	0.14	0.05	0.08	0.18
Brevundimonas	0.01	0.00	0.01	0.01	0.01	0.00	0.01	0.01
Paenibacillus	0.05	0.00	0.00	0.00	0.00	0.00	0.00	0.00
Pseudoxanthomonas	0.04	0.01	0.01	0.01	0.04	0.00	0.01	0.01
Methylobacterium	0.03	0.02	0.01	0.03	0.04	0.01	0.03	0.06
Ruminococcus	0.20	0.08	0.04	0.04	0.38	0.12	0.28	0.23
Streptococcus	0.49	2.72	0.00	0.01	0.15	0.01	0.34	0.24
**Total**	**7.47**	**7.63**	**2.20**	**2.15**	**4.19**	**4.81**	**5.27**	**4.35**
